# CDDO-Me Redirects Activation of Breast Tumor Associated Macrophages

**DOI:** 10.1371/journal.pone.0149600

**Published:** 2016-02-26

**Authors:** Michael S. Ball, Emilie P. Shipman, Hyunjung Kim, Karen T. Liby, Patricia A. Pioli

**Affiliations:** 1 Department of Obstetrics and Gynecology, Geisel School of Medicine, Lebanon, New Hampshire, United States of America; 2 Department of Pharmacology and Toxicology, Geisel School of Medicine, Hanover, New Hampshire, United States of America; Istituto Superiore di Sanità, ITALY

## Abstract

Tumor-associated macrophages can account for up to 50% of the tumor mass in breast cancer patients and high TAM density is associated with poor clinical prognosis. Because TAMs enhance tumor growth, development, and metastatic potential, redirection of TAM activation may have significant therapeutic benefit. Our studies in primary human macrophages and murine breast TAMs suggest that the synthetic oleanane triterpenoid CDDO-methyl ester (CDDO-Me) reprograms the activation profile of TAMs from tumor-promoting to tumor-inhibiting. We show that CDDO-Me treatment inhibits expression of IL-10 and VEGF in stimulated human M2 macrophages and TAMs but increases expression of TNF-α and IL-6. Surface expression of CD206 and CD163, which are characteristic of M2 activation, is significantly attenuated by CDDO-Me. In contrast, CDDO-Me up-regulates surface expression of HLA-DR and CD80, which are markers of M1 activation, and importantly potentiates macrophage activation of autologous T cells but inhibits endothelial cell vascularization. These results show for the first time that CDDO-Me redirects activation of M2 macrophages and TAMs from immune-suppressive to immune-stimulatory, and implicate a role for CDDO-Me as an immunotherapeutic in the treatment of breast and potentially other types of cancer.

## Introduction

Breast cancer is the most commonly diagnosed non-skin cancer among women worldwide and is the second leading cause of cancer death among women [1, 2]. Although early detection and chemotherapeutic treatments have contributed to recent modest declines in breast cancer mortality, the incidence of estrogen receptor negative (ER^-^) breast cancer has not changed significantly in over 30 years and ~40,000 women succumb to breast cancer each year [1]. Thus, there is clearly a pressing need for the development of new, innovative and aggressive approaches to combat this insidious disease.

Numerous studies demonstrate that tumor tissue microenvironments are distinct and consist of malignant as well as non-malignant cell types [3]. Tumor associated macrophages (TAMs) can constitute up to 50% of the tumor mass [3, 4], and high TAM density is correlated with poor clinical prognosis for patients with solid tumors, including breast, prostate, cervical, and ovarian cancers [3, 5]. Genetic depletion of TAMs by conditional M-CSF knockout in mammary epithelium results in a significant delay of tumor progression and inhibition of lung metastasis in the Polyoma virus middle T oncoprotein (PyMT) mouse model of ER^-^ breast cancer, where TAM infiltration is a hallmark feature [6, 7]. These findings suggest that TAM phenotype and function are key factors in enhancing tumor growth.

The tumor microenvironment plays a critical role in shaping TAM activation, as functional skewing of mononuclear phagocytes occurs *in vivo*. Differentiation of monocytes in the unique cytokine milieu of the tumor site polarizes resulting macrophages into M1 (immuno-stimulatory) or M2 (immuno-suppressive) TAMs. In non-progressing or regressing tumors, TAMs resemble classically activated M1 macrophages, as they produce pro-inflammatory cytokines, demonstrate enhanced antigen presentation, and mediate tumor lysis [8]. In contrast, TAMs assume an alternatively activated M2 activation state in malignant tumors, as they suppress adaptive immune responses and secrete anti-inflammatory mediators and angiogenic factors that support tumor growth and metastasis [9]. Because macrophage phenotype and function are plastic and may be redirected by immuno-modulatory cues [10], re-programming TAM polarization from M2 to M1 may have significant therapeutic benefit. While many attempts have been made to redirect TAM activation using cytokines and immune-activators such as LPS [11], these approaches have not been successful for many reasons, including issues with delivery and systemic toxicity. To the best of our knowledge, no drugs have been identified for the treatment of breast cancer that repolarize TAMs.

Triterpenoids are widely used in Asian medicine and include oleanolic acid (OA) and ursolic acid (UA), which have weak anti-inflammatory and anti-carcinogenic properties [12]. CDDO-methyl ester (CDDO-Me), a synthetic oleanane triterpenoid, is >10,000 times more potent in its anti-inflammatory capabilities than its parent OA. This compound has been shown to delay the development of mammary tumors and to arrest the growth of established tumors in the MMTV-neu (mouse mammary tumor virus) transgenic model of ER^-^ breast cancer. In the aggressive PyMT model of ER^-^ breast cancer, recent studies have shown that CDDO-Me not only delays tumorigenesis but also inhibits TAM infiltration of mammary tumors [13]. CDDO-Me is the first drug that is active in this model and with this novel mechanism of action.

We now demonstrate for the first time that in addition to limiting TAM recruitment, CDDO-Me redirects TAMs from M2 polarization to M1. Our studies indicate that CDDO-Me treatment of primary PyMT mammary TAMs inhibits surface expression of M2-characteristic markers and production of anti-inflammatory IL-10 and pro-angiogenic VEGF, and enhances production of M1 surface markers and mediators, including TNF-α. Furthermore, we also show these effects in monocyte-derived M2-skewed human macrophages, suggesting CDDO-Me modulation of TAM activation may not be limited to breast cancer. These results implicate a role for CDDO-Me in the treatment of ER^-^ breast cancer and potentially other types of cancer as well.

## Methods

### Mice

Female heterozygous mice carrying the *Polyomavirus Middle T-Antigen (PyMT)* gene under the control of the MMTV promoter on C57BL/6J background were obtained from Dr. Jeffrey Pollard (Albert Einstein College of Medicine, Bronx, NY). This study was carried out in strict accordance with the protocol (protocol #: liby.kt.1) approved by the Institutional Animal Care and Use Committee (IACUC) of the Geisel School of Medicine at Dartmouth. Mice were euthanized by inhalation of carbon dioxide followed by cervical dislocation. All efforts were made to minimize animal suffering.

### Isolation of Primary PyMT Tumor Associated Macrophages (TAMs)

Mammary tissue was removed from 12-week-old female PyMT mice and incubated in digestion media, which consisted of an enzyme mixture of collagenase (300 U/ml, Sigma), dispase (1 U/ml, Worthington), and DNAse (2 U/ml, Calbiochem), for 45 minutes at 37°C with stirring. Cells were then passed through 70 μm and 40 μm Cell Strainers (BD Falcon), followed by incubation with biotinylated F4/80 antibody (clone: BM8, eBioscience) and a subsequent 15 minute incubation with magnetic streptavidin -coated beads (Miltenyi Biotec). Cells were washed between incubations with PBS (+ 2 mM EDTA). Total F4/80^+^ positive mouse macrophages were isolated according to the manufacturer’s specifications (Miltenyi Biotec) in PBS (+ 2 mM EDTA and 0.5% FBS). Positively selected TAMs and negative flow-through fractions were immuno-phenotyped using flow cytometry of surface markers described in Table 1 to ensure purity of TAM isolation.

**Table 1:** Antibody/fluorophore conjugates used for multi-color flow cytometry. All antibodies were monoclonal and purchased from BioLegend.

Species	Marker	Fluorophore	Clone	Concentration (μg/ml)
**Human**	CD206	FITC	15–2	2
**Human**	CD163	PE	GHI/61	2
**Human**	CD1a	PerCP/Cy5.5	HI149	1.5
**Human**	CD14	PE/Cy7	HCD14	2
**Human**	CD80	APC	2D10	2
**Human**	HLA-DR	APC/Cy7	L243	2

### Human Peripheral Blood Mononuclear Cells (PBMCs) and Generation of Monoctye-derived Macrophages

PBMCs were obtained by leukapheresis of healthy donors following informed consent. Written consent was obtained from all subjects in accordance with the human experimentation guidelines established by Geisel School of Medicine’s Committee for the Protection of Human Subjects, which approved this study (protocol # 17011). Mononuclear cells were separated on Ficoll-Paque Premium (density: 1.077, GE Healthcare) and enriched for monocytes using cold aggregation [14]. Monocyte purity was assessed using cytospin, Wright-Giesma staining and flow cytometric analysis of CD14 expression and was ≥95%.

To generate human macrophages, CD14^+^monocytes were cultured in complete HEPES-buffered RPMI 1640 supplemented with 10% FBS and either 10 ng/ml GM-CSF or 50 ng/ml M-CSF for 7 days, as differentiation with these stimuli results in M1 or M2 macrophage polarization, respectively [15]. Macrophage polarization was verified using flow cytometry to measure expression of cell surface markers CD206 (clone: 15–2), CD163 (clone: GHI/61), and HLA-DR (clone: L243) and by ELISA analysis of secreted cytokines post-LPS stimulation.

### Cell Culture & Reagents

Primary mouse TAMs were cultured in DMEM (4 mM L-glutamine, 4500 mg/L glucose) supplemented with 10% FBS, 0.25 M HEPES, and 100 μg/ml penicillin streptomycin. Human monocytes and macrophages were cultured in RPMI (2.05 mM L-glutamine) supplemented with 10% FBS, 0.25 M HEPES, and 12 μg/ml gentamicin. Human and mouse cells were rested overnight post-isolation and prior to stimulation. For activation studies, cells were pretreated with indicated concentrations of CDDO-Me or DMSO vehicle control for 16 hours, followed by stimulation with 10 ng/ml LPS (Invitrogen, Lot: 33-505-LPS) for an additional 24 hours.

### RNA extraction, cDNA synthesis, and qRT-PCR

Total RNA from human and mouse cells was obtained using the miRNeasy Mini Kit (Qiagen) per manufacturer’s instructions. Complementary DNA (cDNA) was synthesized from 100 ng total RNA and random hexamers using the SuperScript III First-Strand Synthesis System (Life Technologies). Quantitative real time PCR (qRT-PCR) was performed using TaqMan Probe single tube assays (Life Technologies) for human (CCL18, IL-6, IL-10, VEGF, TNF-α, CCR5, CD163, CLEC10A) and mouse (IL-10, Arg1, Ym1, TNF, CXCL9) genes. The StepOnePlus Real-Time PCR System (Applied Biosystems) was used for amplification and detection. Threshold cycle number was determined using Opticon software. mRNA levels were normalized to β-actin, which control studies showed is not altered by CDDO-Me treatment, using the equation 2^-(Et-Rt)^, where Rt is the mean cycle threshold for the control gene and Et is the mean threshold for the experimental gene. Thermal cycling conditions for qRT-PCR consisted of an initial incubation at 50°C for 2 min and 95°C for 10 min, followed by 40 cycles of 95°C for 15 sec and 60°C for 1 min. Product accumulation was measured during the extension phase and all samples were run in triplicate.

### Multi-Plex Cytokine Assay and Enzyme-linked Immunosorbent Assay (ELISA)

TAMs were plated at 2 x 10^5^ cells per well in 24 well tissue culture dishes in complete DMEM. Cells were cultured with vehicle or CDDO-Me for 16 hours followed by stimulation with 10 ng/ml LPS for 24 hours. Cell-free culture supernatants were aliquoted and stored at -80°C until further use. The Milli-Plex suspension array system using fluorescently dyed Luminex microspheres (beads) (EMD Millipore) was used to measure cytokine secretion. This assay system is ideally suited to measure multiple cytokines from one sample. Standards were prepared in the same fresh medium that was used to culture experimental samples and were assayed in triplicate. Spiked controls accurately reflected the added cytokine, chemokine, or growth factor concentration. Assays were performed in a 96 well filtration plate at room temperature according to the manufacturer’s protocol. The fluorescence intensity for each bead was measured using the Bio-Plex array reader. Bio-Plex manager software with five parametric-curve fitting was used for data analysis. The level of detection of each analyte was 7.8 pg/ml. As indicated, supernatants from TAM and human macrophage cultures were analyzed by ELISA (R&D Systems), according to the manufacturer’s protocol.

### Flow Cytometry

All mouse and human fluorophore-conjugated antibodies were obtained from Biolegend and are listed in Table 1. Cell staining was performed for 1 hour at 4°C, with 2 mg/ml Globulins Cohn fraction II, III (Sigma) for human cells or 5 μg/ml anti-mouse CD16/CD32 antibody (clone: 93, eBioscience) for mouse cells to reduce antibody binding to Fc receptors. In all conditions, only adherent cells were stained following cell culture, and doublets were excluded by FSC-A vs. FSC-H gating. Gating of positively stained cells was determined by fluorescence-minus-one (FMO) controls, represented as black histograms. Cells were analyzed using an 8-color MACSQuant 10 (Miltenyi Biotec) with three laser sources (405 nm, 488 nm, 635 nm) and FlowLogic 501.2A software (Inivai Technologies.

### CFSE Proliferation Assay

Human monocytes were isolated from PBMCs derived from healthy donors as above. Post-monocyte isolation, flow-through fractions were stained with 2.5 μM CFSE using CellTrace™ CFSE Cell Proliferation Kit according to the manufacturer’s protocol (Life Technologies). CFSE-stained cells were stimulated in the presence of 2.5 μM soluble αCD3 and 1 μM soluble αCD28 to stimulate T cell proliferation and cultured for 4 days with autologous LPS-stimulated M2 macrophages that had been treated +/- 300 nM CDDO-Me for 16 hours or M1 macrophages. Percent of T cell proliferation was determined by CFSE dilution using flow cytometry. Data were analyzed using FlowLogic 501.2A (Inivai Technologies).

### Angiogenesis Tube Formation Assay

Human umbilical vein endothelial cells (ECs; Lonza) were cultured in Endothelial Growth Medium (EGM-2, Lonza) to between 65% and 80% confluence (passage < 3). Cells were dissociated with 0.05% trypsin (Hyclone) and plated in a 96-well plate containing reduced growth factor basement membrane extract supplied with the In Vitro Angiogenesis Tube Formation Assay Kit (Trevigen). Cells were then cultured at 37°C for 10 hours in the presence of EGM-2, endothelial basal media (EBM-2, Lonza) without serum or supplements, or conditioned media from TAMs treated with DMSO control or 300 nM CDDO-Me. Tube formation was visualized using an Olympus IX73 inverted fluorescence microscope. Quantification was performed using AngioTool software to determine total number of cellular junctions, as previously described [16].

### Statistical Analysis

**Fig 1:**
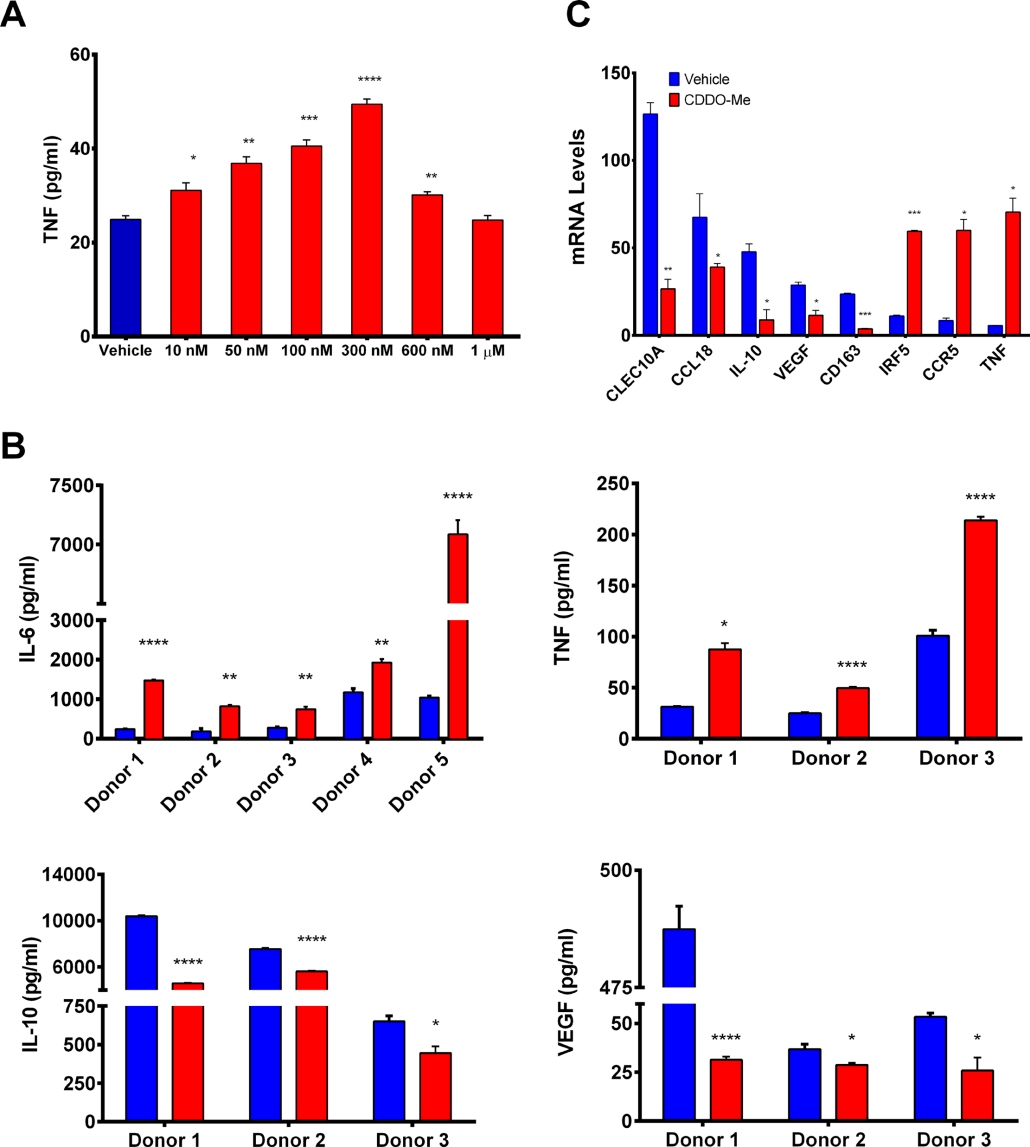
CDDO-Me redirects LPS-induced cytokine production of human M2 macrophages. Human peripheral blood-derived monoctyes were differentiated with M-CSF to generate alternatively activated (M2-polarized) macrophages. (A) CDDO-Me dose-dependently induced TNF-α secretion in LPS-stimulated macrophages. Culture supernatants from macrophages treated with CDDO-Me or DMSO control for 16 hrs prior to stimulation with LPS (10 ng/ml) for 24 hrs were analyzed by ELISA for TNF-α. Data are representative of results obtained with 3 individual donors, and depict average values obtained from 3 independent assays. (B) ELISA analysis of IL-6, IL-10, TNF-α and VEGF secretion from human macrophages pretreated with 300 nM CDDO-Me for 16 hrs followed by activation with LPS as in (A). CDDO-Me effects on IL-6 secretion were assayed in 5 donors; 3 donors were assayed for effects on TNF, IL-10, and VEGF. (C) Total RNA was extracted from macrophages pretreated with or without CDDO-Me (300 nM) for 16 hrs followed by LPS for 24 hrs. mRNA transcript levels were measured by Taqman real time PCR *p<0.05, **p<0.01, ***p<0.005, ****p<0.001 vs. untreated control. Data shown are representative of results obtained from analysis of 3 individual donors. CDDO-Me effects on mRNA levels were analyzed in 3 separate experiments for each donor. As indicated in Methods, 3 technical replicates were analyzed in each separate, individual experiment.

**Fig 2:**
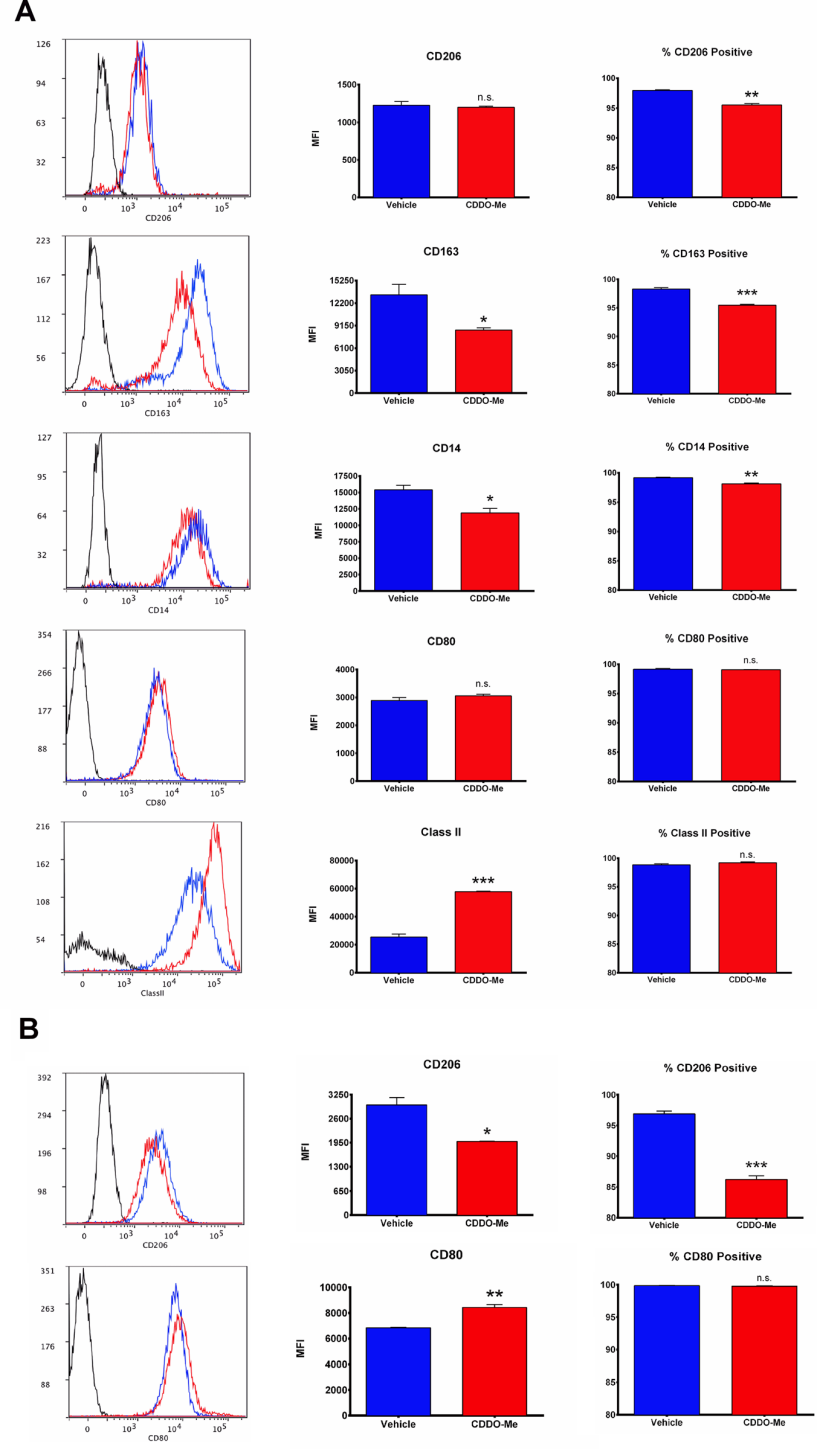
CDDO-Me up-regulates surface expression of M1-characteristic markers and attenuates expression of M2 markers. Human M2-polarized macrophages were treated with 300 nM CDDO-Me or DMSO control for 16 hrs (A) without or (B) with stimulation with LPS for 24 hrs. Expression levels of surface markers were quantified using flow cytometric analysis and are presented in mean fluorescence intensity (MFI) units. Gating of positively stained cells was determined by FMO controls (black histograms). *p<0.05, **p<0.01, ***p<0.005 vs. untreated control. Data are representative of results obtained with 3 individual donors. Graphs indicate average values obtained from 3 independent assays. In addition, 3 technical replicates were performed for each assay. Error bars represent experimental standard error of the mean (SEM).

**Fig 3:**
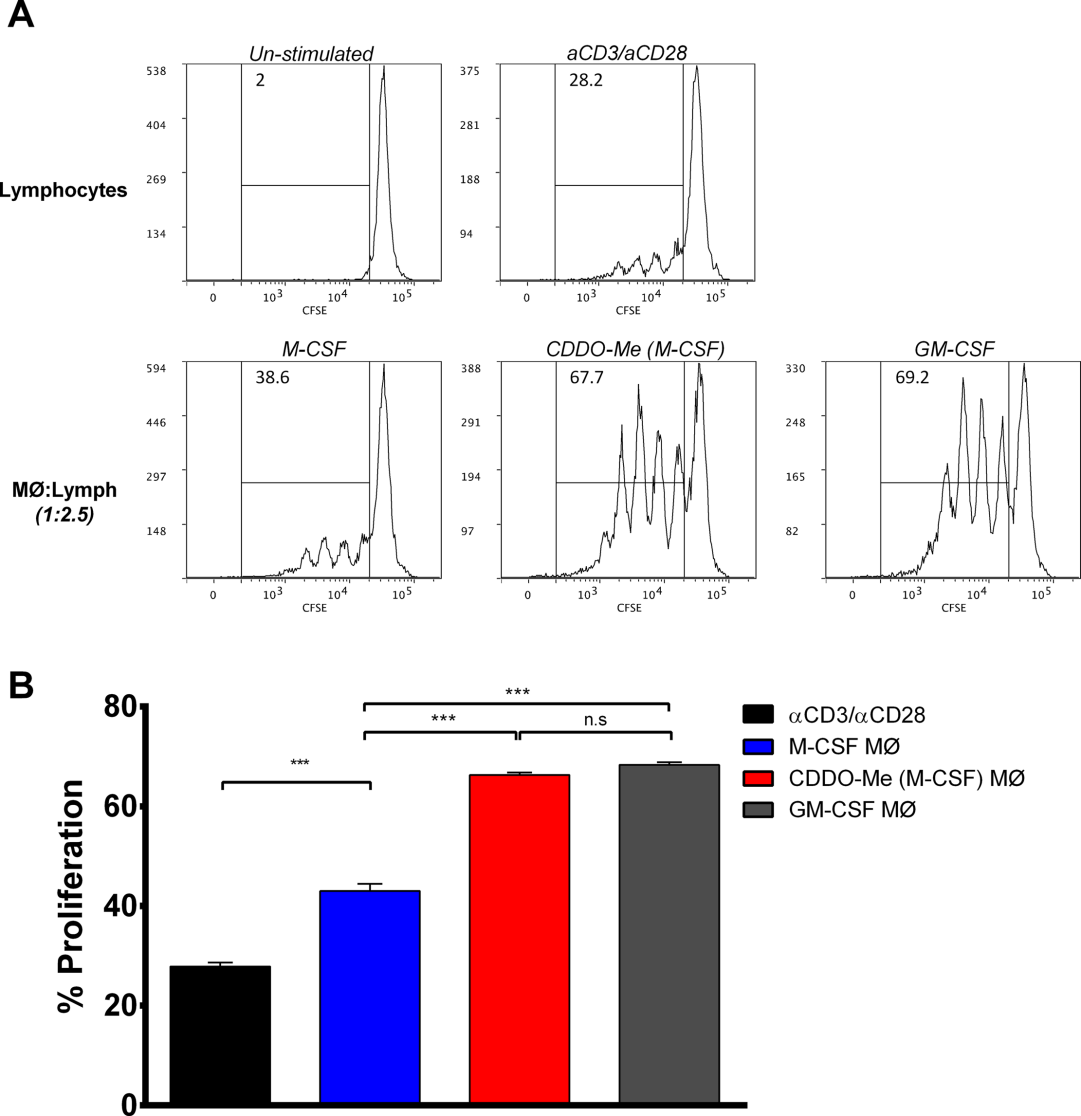
CDDO-Me treatment enhances the ability of M2 macrophages to induce T cell proliferation. (A) Representative histograms of CFSE proliferation assay. Top panels show un-stimulated and stimulated (αCD3/αCD28) T cell proliferation in media alone. In bottom panels, macrophages were differentiated with GM-CSF to generate M1 macrophages or M-CSF to elicit M2 macrophages. M2 macrophages were treated +/- CDDO-Me for 16 hrs followed by LPS stimulation for 24 hours and co-cultured with autologous T cells. T cell proliferation was measured by CFSE dilution using flow cytometry. (B) Graphical depiction of representative CFSE experiment. Data are representative of results obtained from 3 independent proliferation assays, and 3 technical replicates were analyzed in each assay. ***p<0.005; biological replicates, n = 2.

**Fig 4:**
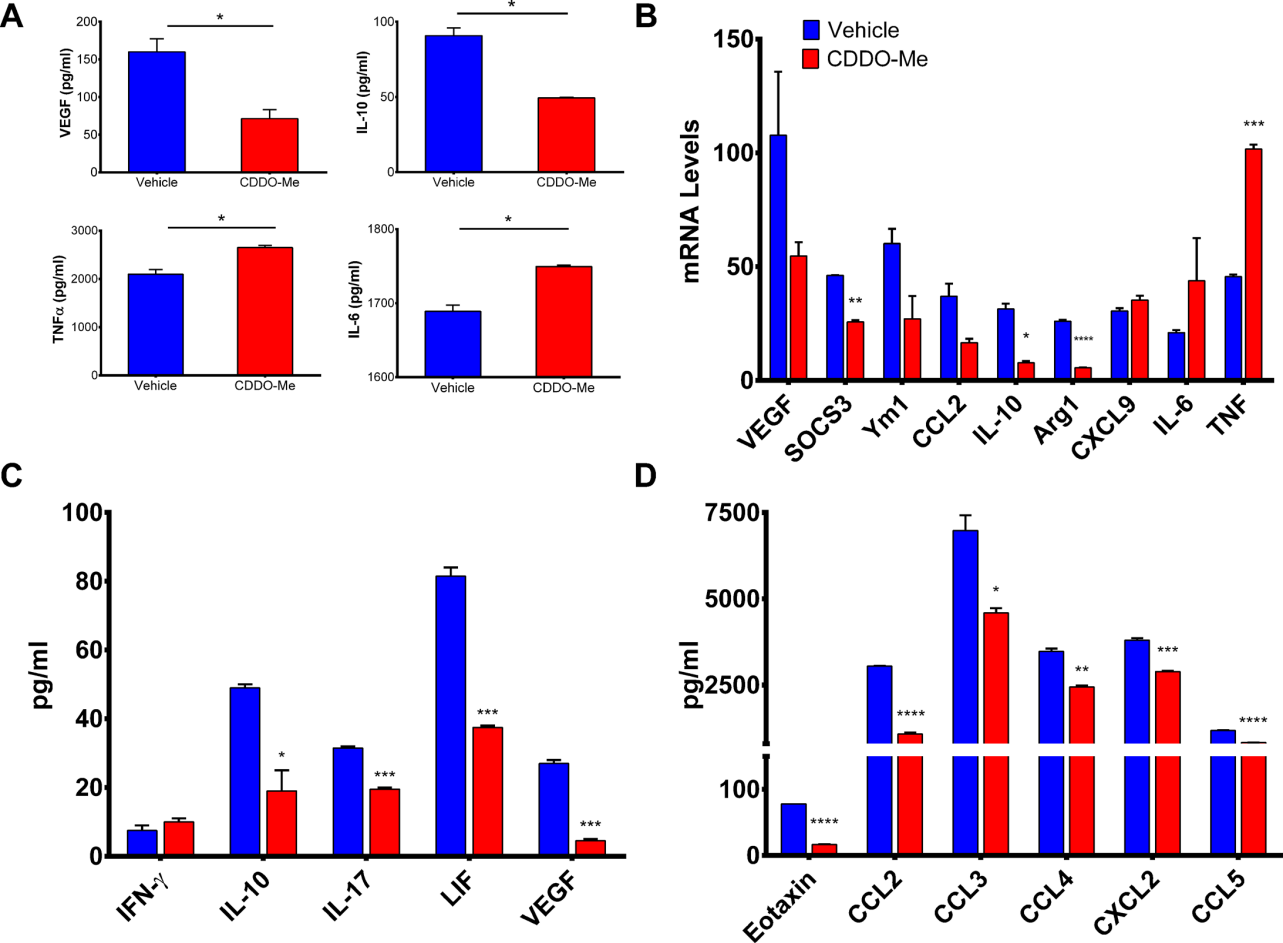
TAM activation profile is altered by CDDO-Me. F4/80+ tumor associated macrophages (TAMs) were isolated from the tumors of 12-week old female PyMT mice and treated *in vitro* with CDDO-Me (300 nM) for 16 hrs followed by stimulation with LPS (10 ng/ml) for an additional 24 hrs. Cell supernatants were analyzed by (A) ELISA to measure secreted protein levels. (B) Total RNA was isolated from TAMs +/- CDDO-Me. mRNA transcripts levels were measured by Taqman real time PCR. Multiplex analysis was used to quantify expression of cytokines and growth factors (C) and chemokines (D) in CDDO-Me-treated TAM supernatants. *p<0.05 vs. untreated control. (A-D) Data are representative of 3 independent experiments, and 3 technical replicates were analyzed in each experiment.

**Fig 5:**
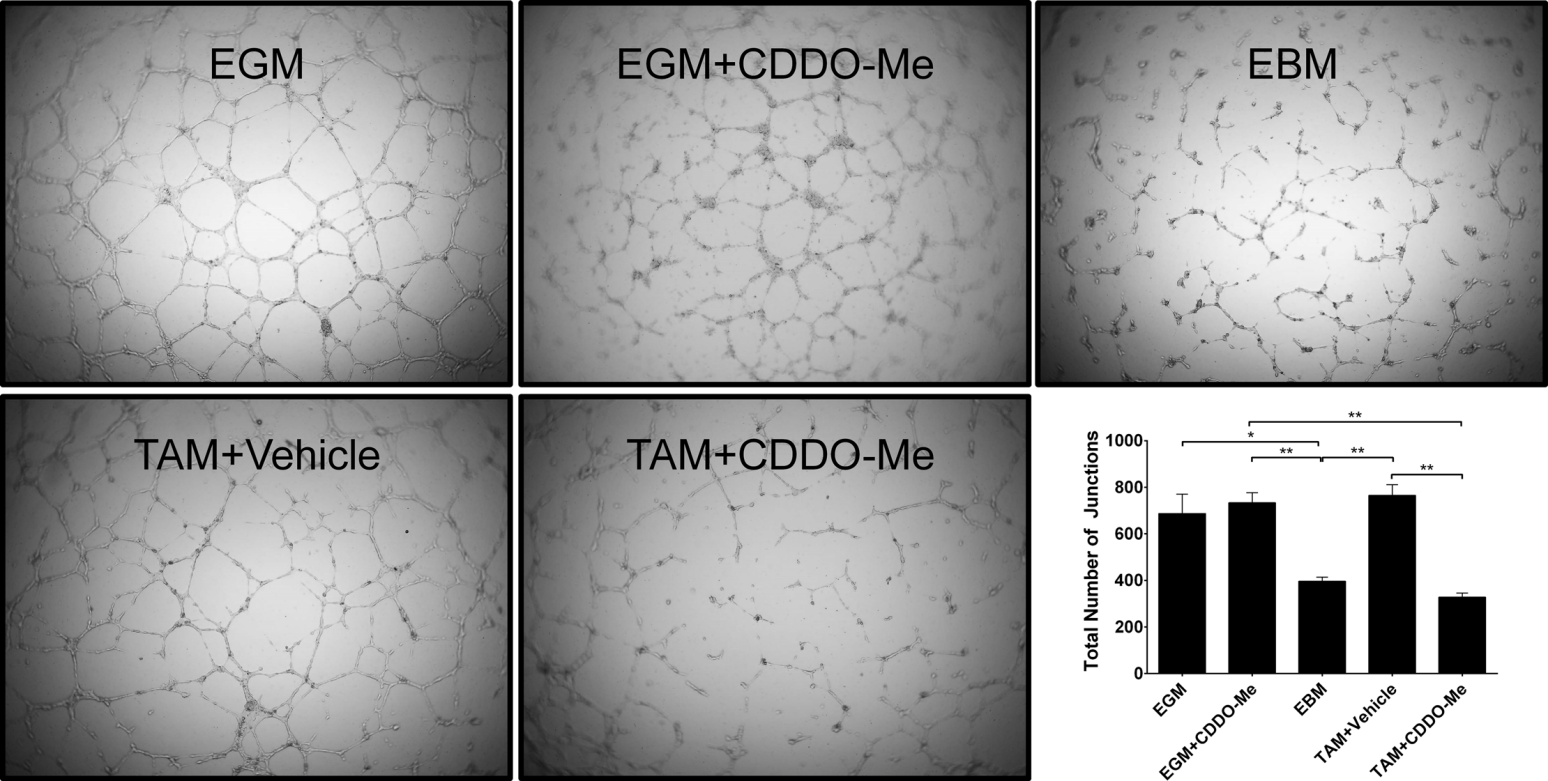
Altered TAM polarization by CDDO-Me inhibits EC vessel formation. F4/80+ tumor associated macrophages (TAMs) were isolated from the tumors of 12-week old female PyMT mice and treated *in vitro* with DMSO control or CDDO-Me (300 nM) for 16 hrs. Conditioned media (CM) were collected from TAM cultures. ECs were cultured on basement membrane extract in the presence of complete endothelial growth media (EGM; top left), endothelial basal media (EBM; top right), endothelial growth media in the presence of CDDO-Me (EGM+CDDO-Me; top center) vehicle-treated TAM CM (TAM+Vehicle, bottom left), or CDDO-Me treated TAM CM (TAM+CDDO-Me, bottom center). Total number of junctions was quantified by AngioTool software and is represented in the bottom right panel. Data are representative of 2 independent experiments. *p<0.05, **p<0.01. Quantification represents mean ± standard deviation.

Figures are representative of three independent experiments, and in human studies, 3–4 different donors were used, as indicated in Figure Legends. All experiments were repeated at least 3 times, unless otherwise noted and at least 3 technical replicates of each analyte were included in each assay. Results in Figs 1–4 are described as mean ± SEM and were analyzed by unpaired student’s t-Test. Significance was achieved at p<0.05. Quantification of results in Fig 5 is represented by mean ± standard deviation.

## Results

### CDDO-Me alters cytokine mRNA levels and protein secretion in human M2 macrophages

While the cytotoxic effects of CDDO-Me on tumor cells have been well-documented, the ability of this drug to modulate activation of immune cells in the tumor microenvironment, including TAMs, is unknown. TAMs promote tumor progression through the elaboration of immuno-suppressive cytokines, chemokines, and angiogenic factors, which are characteristic of M2-polarized macrophages [17, 18]. Because TAMs phenotypically and functionally resemble M2-skewed macrophages, our initial studies were performed using human peripheral blood-derived monocytes with M-CSF to recapitulate this phenotype. To determine the effect of CDDO-Me on TNF-α production in human M2-polarized macrophages, cells were pretreated with CDDO-Me at concentrations ranging from 10 nM-1 μM for 16 hours. Following CDDO-Me treatment, macrophages were activated with LPS for an additional 24 hours, and soluble TNF levels were measured in culture supernatants. As demonstrated in Fig 1A, 300 nM CDDO-Me maximally induced TNF-α production. TNF-α protein levels were undetectable in unstimulated cells in the presence or absence of CDDO-Me. Cell viability was assayed in tandem using the Cell Titer Blue reagent, which demonstrated that CDDO-Me is not toxic to TAMs at concentrations lower than 1μM.

Because CDDO-Me enhanced LPS-induced TNF-α secretion, which is associated with M1 activation, we investigated the effect of CDDO-Me on the expression of IL-10 and VEGF, which are tumor-promoting factors produced by TAMs [19, 20]. As seen in Fig 1B and 1C, CDDO-Me significantly attenuated mRNA and protein expression of both immuno-suppressive IL-10 and angiogenic VEGF. In accordance with our findings in Fig 1A, CDDO-Me augmented production of immuno-stimulatory TNF-α and IL-6 (Fig 1B and 1C) [21]. Notably, although CDDO-Me also enhanced mRNA levels of TNF-α, no significant differences in IL-6 mRNA expression were observed with drug treatment (data not shown), suggesting CDDO-Me regulates IL-6 protein expression post-transcriptionally. As these results suggested CDDO-Me redirects macrophage activation from M2 to M1, CDDO-Me effects on M1 and M2 markers of macrophage activation were assessed. Consistent with earlier results, CDDO-Me inhibited mRNA expression of CD163 and CLEC10A, which are markers of M2 activation [22, 23], but enhanced mRNA levels of IRF5, a transcription factor that represses production of IL-10 [24] and has been shown to be an independent marker of inflammatory macrophages [25] and the chemokine receptor CCR5, which is highly expressed on M1 macrophages [26]. CDDO-Me also decreased mRNA expression of CCL18, which has been implicated in promoting breast cancer metastasis and is highly expressed by human TAMs and M2-activated macrophages [27, 28].

### CDDO-Me attenuates M2 surface marker expression on human macrophages and enhances macrophage T-cell proliferation capabilities

To more extensively interrogate the effects of CDDO-Me on macrophage activation, surface expression of M1 and M2-characteristic markers was measured using multicolor flow cytometry. As shown in Fig 2A, constitutive expression of the hemoglobin/haptoglobin scavenger receptor CD163 and the LPS co-receptor CD14 was inhibited by CDDO-Me treatment. Notably, these surface markers are highly expressed on M2 macrophages and human breast TAMs [29], and tumor expression of CD163 is directly correlated with early distant recurrence and reduced patient survival [30]. Because M1 macrophages have enhanced antigen presentation and T lymphocyte activation capabilities compared with M2 cells [31], we next evaluated CDDO-Me effects on surface expression of MHC-II and the co-stimulatory molecule CD80. In accordance with CDDO-Me-mediated redirection of macrophage activation, surface levels of MHC-II increased three-fold, and although not sufficient to reach significance, CD80 levels rose by 15 percent (Fig 2A). As reports have shown that expression of CD80 and CD206 is LPS-inducible [32, 33], we also assessed CDDO-Me-mediated effects on these markers in the context of LPS stimulation. Cells pretreated with CDDO-Me and subsequently stimulated with LPS upregulated CD80 expression by 20 percent and significantly reduced CD206 positivity (33 percent) (Fig 2B). Evaluated in aggregate, results of immuno-phenotyping demonstrate that CDDO-Me significantly attenuates immuno-suppressive M2 activation.

To establish whether CDDO-Me altered macrophage function as well as cytokine production and surface marker expression, we evaluated the ability of CDDO-Me-treated TAMs to stimulate T cell proliferation. Human M1 or M2 macrophages treated with or without CDDO-Me were cultured with autologous lymphocytes labeled with CFSE (Fig 3). T-cells were stimulated with soluble αCD3 and αCD28 to induce activation and cultured in the presence or absence of macrophages for four days. As predicted, M1 (GM-CSF-differentiated) macrophages significantly enhanced T cell proliferation compared with M2 (M-CSF-differentiated cells). However, M2 macrophages treated with CDDO-Me stimulated T cell proliferation at levels comparable to M1 cells (Fig 3). Thus, CDDO-Me modulation of M2 activation enhances mobilization of adaptive immune responses and skews them toward an M1-like activation state.

### Immuno-suppressive cytokine production in PyMT TAMs is markedly attenuated by CDDO-Me

Because CDDO-Me redirected activation of human M2-polarized macrophages, we next asked whether CDDO-Me might mediate similar effects on TAMs. To determine this, we utilized the PyMT mouse model of ER^-^ breast cancer, as we have previously shown that *in vivo* treatment with CDDO-Me delays mammary tumor onset and progression in this model, concurrent with decreased recruitment of TAMs [13]. Another advantage of this model is that it closely mimics the progression of human disease and PyMT tumors are characterized by a high myeloid infiltrate [34]. F4/80^+^ TAMs were isolated from the mammary glands and tumors of 12 week old PyMT mice and cultured with 300 nM CDDO-Me. As demonstrated in Fig 4A and 4C, CDDO-Me inhibited secretion of VEGF by 65 percent and IL-10 by 45 percent but significantly increased TNF-α and IL-6 protein expression; these changes were also reflected in altered mRNA levels (Fig 4B). As in our studies of human macrophages, expression of M2-characteristic markers was consistently attenuated by CDDO-Me (Arg1, Ym1, CCL2, and SOCS3), while expression of M1 markers was enhanced (CXCL9, TNF-α, and IL-6) (Fig 4B) [10, 35]. Intriguingly, CDDO-Me elicited a small increase in TAM production of IFN-γ(Fig 4C), which is notable as M-CSF-differentiated macrophages have been reported to secrete IFN-γin the presence of pro-inflammatory IL-12 [36]. TAM production of LIF, which has been shown to mediate M2 polarization of TAMs [37], was attenuated by CDDO-Me treatment, as was production of IL-17, which has been linked to poor prognosis in breast cancer [38].

CDDO-Me also inhibited expression of chemokines that mediate selective recruitment of TAMs and M2 macrophages, (CCL3, CCL4, and eotaxin) [39–41] and monocytic infiltration of tumor sites (CCL2 and CCL5) [42] (Fig 4D). Notably, we have previously reported CDDO-Me-mediated down-regulation of CCL2 expression in PyMT tumor cells [13]. CXCL2, which regulates breast cancer metastasis and chemo-resistance [43], is also reduced by CDDO-Me treatment (Fig 4D), implicating a potential role for CDDO-Me in the treatment of metastatic disease. Collectively, these results suggest CDDO-Me attenuates immune-suppressive TAM activation in favor of an enhanced immuno-stimulatory TAM activation profile.

### CDDO-Me-redirection of TAMs inhibits endothelial cell (EC) tube formation

High VEGF production and subsequent increased microvasculature in tumors are associated with TAM infiltration in breast cancer, and are negative prognostic indicators for relapse and survival in patients [40, 44]. Because CDDO-Me markedly attenuated VEGF production by TAMs (Fig 4A and 4B), we hypothesized that CDDO-Me-redirected TAMs would inhibit EC angiogenesis. To test this, ECs were cultured with conditioned media from TAMs treated with vehicle or 300 nM CDDO-Me and plated in a tumor-derived basement membrane extract. Under permissive conditions, ECs in this matrix will organize into structures that resemble microvessels or tubes. As demonstrated in Fig 5, ECs incubated with conditioned media from vehicle-treated TAMs (DMSO) formed tubular structures with several junctions between adjacent cells, similar to cells cultured with complete endothelial growth media (EGM). In contrast, ECs cultured with CDDO-Me- treated TAM conditioned media (300 nM CDDO-Me) did not form discrete tubes, consistent with cells cultured in endothelial basal media (EBM). These in vitro studies establish that CDDO-Me affects ECs indirectly via TAM polarization, as culture of ECs with CDDO-Me directly did not affect EC proliferation, viability or tube formation (EGM+CDDO-Me). These results demonstrate that CDDO-Me inhibits EC vessel formation via indirect effects on TAM polarization rather than direct effects on ECs.

## Discussion

This study demonstrates for the first time that the synthetic triterpenoid CDDO-Me markedly attenuates the immunosuppressive activation state of primary breast TAMs, inhibiting both RNA and protein expression of tumor-promoting IL-10 and VEGF, while enhancing expression of immune-stimulatory TNF-α, IFN-γ, and IL-6. In addition, we also demonstrated using human M2-polarized macrophages that CDDO-Me decreases expression of cytokines and surface markers associated with M2 activation and concomitantly increases markers of M1 activation. Because we have shown that CDDO-Me mediates these effects in M2-skewed macrophages that recapitulate TAM activation, we believe this drug may modulate TAM polarization in other types of cancer as well. These results provide the first evidence that CDDO-Me is capable of redirecting TAM activation from an immune-suppressive to an immune-activating state.

Although we and others have extensively documented the direct cytotoxic effects on CDDO-Me on tumor cells (reviewed in [12]), immune function is important for CDDO-Me inhibition of tumor growth, as tumors grown in an injectable model of SCID mice that lack functional lymphocytes show no response to CDDO-Me treatment [45]. Moreover, work by Nagaraj *et al*. demonstrates that CDDO-Me inhibits activation of myeloid-derived suppressor cells (MDSCs) through blockade of ROS production [45], implicating innate immune mediators as additional targets of CDDO-Me. In this regard, we have shown that CDDO-Me impedes TAM recruitment to tumors in PyMT mice through inhibition of tumor cell production of CCL2 and CXCL12 [13]. This finding, coupled with chemokine data from our current study, implicates CDDO-Me as a regulator of myeloid cell migration and activation. Notably, we detected a marked decrease in surface expression of CD163, which is characteristic of M2 macrophages and TAMs and is associated with poor clinical prognosis [46, 47]. Previous work has demonstrated that CD163 is upregulated by IL-10 [48] and that IL-10 induces M2 activation of macrophages [49]. As we have now shown that IL-10 production is inhibited by CDDO-Me, it is therefore possible that this provides a negative feedback mechanism for diminished expression of CD163 and redirection of TAM activation. In this regard, we noted that CDDO-Me-mediated attenuation of another M2-associated maker, CD206, was greatly amplified under conditions of cellular activation. It is possible that CDDO-Me modulates expression of CD206 in these conditions by both direct and indirect mechanisms. Our findings suggest that CDDO-Me may modulate expression of an LPS-inducible regulator of CD206, which may account for the difference in the magnitude of the effect under basal and stimulated conditions. One potential candidate is GM-CSF, which has been reported to induce CD206 expression [50], and which we have shown is inhibited by CDDO-Me (unpublished observations). Experiments are currently underway to test this hypothesis. Furthermore, because CDDO-Me is pleiotropic, the molecular mechanism(s) by which CDDO-Me regulates TAM activation are likely to be multifactorial. Candidate pathways include NFκB, STAT3, and PPARγ, as each of these is modulated by CDDO-Me [51–53] and has been shown to regulate macrophage polarization [54–56].

While this work has focused on direct drug effects on TAM activation, CDDO-Me may mediate indirect effects on TAM polarization through modulation of tumor cell-secreted factors. In this regard, tumor cells have been shown to direct TAM polarization through release of cytokines including IL-4 and TGF-β[57]. Indeed, tumor cell apoptosis has been implicated in enhancing immune-suppressive breast TAM activation [58], and CDDO-Me induces tumor cell apoptosis in some cancer models [12]. Thus, it will be important to define the contribution of CDDO-Me-modulated tumor cells to TAM polarization.

Angiogenesis plays a significant role in tumor growth and progression to metastatic disease, and TAMs contribute to vessel formation in a phenotype-dependent manner [59]. Since CDDO-Me redirection of TAM activation significantly impaired endothelial cell tube formation, this drug may be important therapeutically in eliciting both immune activation and in combatting angiogenesis. In accordance with recent findings [60], we failed to observe direct effects of CDDO-Me on EC viability. Furthermore, our studies suggest CDDO-Me effects on EC vascularization are likely mediated indirectly though changes in TAM polarization, rather than through direct effects on ECs, as we did not observe changes in EC tube formation in the absence of CDDO-Me TAM conditioned media. These findings differ from a previous observation [61], but may be attributable to differences in drug timing and dose. Notably, these authors also reported that CDDO-Me suppressed angiogenesis much more potently *in vivo* compared with *in vitro*, suggesting that CDDO-Me anti-angiogenic activity is likely the result of drug effects on multiple cell types within the tumor microenvironment. Nevertheless, our studies demonstrate that CDDO-Me-mediated changes in TAM activation influence EC angiogenesis. We speculate that CDDO-Me induces these changes through downregulation of M2 angiogenic cytokines (VEGF and IL-10). Our results imply that re-education of TAM polarization is a promising anti-angiogenic approach, and stress the importance of analyzing TAM function as well as numbers.

It is imperative to note that while the M1/M2 designation is useful in assigning immune-suppressive and immune-enhancing properties to macrophages, it is somewhat limited in its utility to accurately describe macrophage activation. Because macrophages are plastic and subject to modulation by local micro-environmental factors, *in vivo* macrophage activation spans a broad spectrum of polarization states [62]. We selected M-CSF to mediate M2 activation of human macrophages, as tumor cell-derived M-CSF has been shown to drive the differentiation of pro-angiogenic TAMs in breast cancer [63]. However, because the tumor microenvironment is diverse and replete with many other potential immune-modulatory factors, it will be important to verify the effects of CDDO-Me on TAM activation *in vivo*. As such, experiments to address *in vivo* regulation of TAM activation by CDDO-Me are ongoing. Nevertheless, it is notable that we were able to recapitulate CDDO-Me effects on primary TAMs in human monocyte-derived macrophages. These findings suggest our observations in the PyMT model may be potentially extrapolated to other types of cancer.

Because inhibition of IL-10 has been shown to mediate enhanced responsiveness to chemotherapy [64], CDDO-Me treatment may also be beneficial in increasing the efficacy of other chemotherapeutic agents. TAMs are the dominant source of IL-10 in mammary carcinomas [65], and recent work demonstrates that TAM-derived IL-10 blocks CD8^+^ T cell-dependent responses to chemotherapy by suppression of IL-12 expression by intra-tumoral dendritic cells [64]. Although we were unable to detect direct changes in IL-12 expression by CDDO-Me-treated TAMs *in vitro*, our preliminary studies suggest that *in vivo* administration of CDDO-Me does elicit IL-12 production from PyMT TAMs (data not shown). It is also entirely possible these effects are mediated indirectly by DCs. Current studies in our laboratory are focused on optimizing the efficacy of combination therapies utilizing CDDO-Me and other chemo and immuno-therapeutics to halt tumor growth and progression.

The immunosuppressive tumor microenvironment not only fosters tumor progression but has also proven a major impediment to the efficacy of cancer immunotherapies. Because TAMs constitute the dominant myeloid cell population in mammary tumors and are a major source of immunosuppression, altering TAM activation may provide a means of alleviating this barrier. Although previous attempts have been made to modulate TAM function using cytokines and immune activators such as LPS, these approaches have been hampered by issues associated with delivery and substantial toxicity [11, 66]. This study is the first to demonstrate that CDDO-Me, an orally available drug that is well-tolerated in cancer patients [67], redirects TAM activation. We also show that CDDO-Me stimulates M2-polarized macrophages to induce proliferation of autologous T cells to levels that are comparable to M1-activated cells. Furthermore, CDDO-Me-redirected TAMs also inhibit EC vessel organization. The potent immuno-stimulatory and anti-angiogenic activity of CDDO-Me, coupled with its direct anti-proliferative effects on tumor cells, suggests that this drug may be an important component in our arsenal of immuno-therapeutics to combat cancer.
